# Patient Safety in the Process of Pharmacotherapy Carried Out by Nurses—A Polish–Slovak Prospective Observational Study

**DOI:** 10.3390/ijerph181910066

**Published:** 2021-09-25

**Authors:** Izabela Witczak, Łukasz Rypicz, Mária Šupínová, Elena Janiczeková, Piotr Pobrotyn, Agnieszka Młynarska, Olga Fedorowicz

**Affiliations:** 1 Department of Health Care Economics and Quality, Faculty of Health Sciences, Wroclaw Medical University, 50-367 Wroclaw, Poland; izabela.witczak@umed.wroc.pl; 2 Faculty of Health, Slovak Medical University, 947-05 Bratislava, Slovakia; maria.supinova@szu.sk (M.Š.); elena.janiczekova@szu.sk (E.J.); 3 University Teaching Hospital, 50-556 Wroclaw, Poland; dn@usk.wroc.pl; 4 Department of Gerontology and Geriatric Nursing, Faculty of Health Sciences in Katowice, Medical University of Silesia, 40-055 Katowice, Poland; amlynarska@sum.edu.pl; 5 Department of Clinical Pharmacology, Faculty of Pharmacy, Wroclaw Medical University, 50-556 Wroclaw, Poland; olga.fedorowicz@umed.wroc.pl

**Keywords:** patient safety, pharmacotherapy process, risk factor, nurse

## Abstract

Pharmacotherapy, i.e., the use of medicines for combating a disease or its symptoms, is one of the crucial elements of patient care. Nursing workloads in the pharmacotherapy process prove that nurses spend 40% of their work on the management of medications. This study was aimed at the determination and comparison of safety levels at the nurse-managed stage of the pharmacotherapy process in Poland and Slovakia by identifying the key risk factors which directly affect patient safety. The study involved a group of 1774 nurses, of whom 1412 were from Poland and 362 were from Slovakia. The original Nursing Risk in Pharmacotherapy (acronym: NURIPH) tool was used. The survey questionnaire was made available online and distributed to nurses. The Cronbach’s alpha coefficient was 0.832. Nurses from Slovakia most often, i.e., for six out of nine factors (items: one, five, six, seven, eight, and nine), assessed the risk factors as “significant risk (3)”, and Polish nurses most often, i.e., for as many as eight out of nine risk factors (items: one, two, three, four, five, six, seven, and nine), assessed the risk factors as “very significant (5)”. It has been found that the safety of the pharmacotherapy process is assessed by Polish nurses to be much lower than by Slovak nurses.

## 1. Introduction

Patient safety is a relatively new research trend; it nonetheless arouses great interest of researchers. The World Health Organization (WHO), for nearly a decade, has undertaken numerous actions to promote patient safety, including the promotion of medication without harm. In 2020, the WHO declared their Flagship Initiative “A Decade of Patient Safety 2020–2030”. All the more so, the research area explored by the authors of this article proves immensely important, taking into account the scarcity of publications on the detailed analysis of risk factors in the pharmacotherapy process. Tools that could be used for this purpose are also scarce.

Pharmacotherapy, i.e., the use of medicines for combating a disease or its symptoms, is one of the crucial elements of patient care. The proper course of pharmacotherapy has an impact on patient recovery, the alleviation of symptoms and improvements in the health and quality of life of patients. The administration of medications is of essential importance for patient safety, and medication administration errors (MAEs) are directly related to mortality and morbidity rates [1,2]. Studies on nursing workloads in the pharmacotherapy process prove that nurses spend 40% of their work on the management of medication [3]. Keohane et al. note that medicine administration is the most frequent task of all nursing activities, and there is a possibility of committing errors at this stage due to the complex and multifaceted nature of the pharmacotherapy process [4].

The dynamics of a safe medicine administration process can be disturbed by the number of patients per nurse, their clinical condition and comorbidities, fatigue and stress of the nursing staff, inadequate working conditions, and disturbances in the communication in the interdisciplinary team involved in the medicine management process [5,6,7].

It has been revealed that the most frequent errors in the pharmacotherapy process are the prescription of medicines (46%) and medicine administration (41%) [8].

Dean B.S. et al. in their study aimed to formulate a definition of prescribing errors, made a distinction from cognitive errors (related to the decision-making by physicians, for example, prescribing medication for a patient for whom that medication is contraindicated) and technical errors, such as an illegible order, using abbreviated medication names, entering “milligrams” instead of “micrograms”, etc. [9].

Nurses can contribute to technical errors, and the prevention of such errors depends not only on the professionalism of nurses, but also on organizational factors and the work environment. There are numerous alarming risk factors that contribute to the occurrence of errors in nursing practice. The most significant of them include burnout, understaffing and heavy workload [10,11,12]. In order to prevent errors in the pharmacotherapy process, nurses have to use strategies such as the discrimination of high-risk medications, separating medications with similar names, error analyses, and increasing the awareness of medication errors [13,14]. 

This study aimed to determine and compare safety levels at the nurse-managed stage of the pharmacotherapy process in Poland and Slovakia by identifying the key risk factors which directly affect patient safety.

## 2. Materials and Methods

### 2.1. Design and Settings

The study was conducted from 1 May 2019 to 30 September 2019. During this time, data were collected in collaboration with the Supreme Chamber of Nurses and Midwives in Poland and the Slovak Chamber of Nurses and Midwives. In Poland, messages were sent to the District Chambers of Nurses and Midwives with information about surveying pharmacovigilance safety among nurses. The invitation to participate in the study was accompanied by a link to the website where the questionnaire could be answered. In Slovakia, the distribution of the test link was similar. The link to the questionnaire was sent by e-mail via the central register of nurses of the Slovak Chamber of Nurses and Midwives.

### 2.2. Data Collection 

The criteria for inclusion in the study were the possession of a valid license to practice as a registered professional nurse and documented professional activity. The study involved 1774 participants from all over Poland and Slovakia, including 1412 nurses from Poland and 362 nurses from Slovakia. The survey excluded 467 subjects who did not complete the questionnaire; thus, the results were not completed and, as such, rejected from further analysis. 

The NURIPH—Nursing Risk in Pharmacotherapy questionnaire (Witczak et al., 2020) [15] was available on the website throughout the survey. In Poland, the link to the questionnaire was published on the website of the Supreme Chamber of Nurses and Midwives and the website of the District Chambers of Nurses and Midwives. In Slovakia, the link was sent by e-mail via the central register of nurses of the Slovak Chamber of Nurses and Midwives. Additionally, the study was promoted during national conferences dedicated to nurses. Each participant completed an anonymous NURIPH questionnaire via the internet. After completing the questionnaire, participants confirmed and sent the questionnaire electronically to the platform where the data were collected.

### 2.3. Research Tool

The original Nursing Risk in Pharmacotherapy (NURIPH) tool was used. NURIPH’s proprietary tool consists of three parts: a metric for socio-demographic data, a risk matrix for assessing selected ergonomic factors that cause risk in the pharmacotherapy process, and questions about the organization’s safety culture and nurses’ opinions on issues related to medication errors (questions outside the matrix are summarized in section: Results. Nine risk factors were considered in the matrix: 1—Poorly legible or illegible medical orders; 2—Inappropriate communication between physician, nurse, and midwife regarding changes in drug orders; 3—Time pressure during nurse/medical supervision; 4—Inappropriate work organization: preparation of medicines for patients combined with the simultaneous performance of other activities by a nurse/midwife (for example, answering calls, execution of current diagnostic orders, etc.); 5—Lack of clarity or illegibility of medical orders for nurses and midwives; 6—No physician’s prescription of a specific solvent for a particular medicine; 7—Shift work causing psycho-physiological fatigue; 8—Limited availability of training on the effects of medicines, side effects and adverse reactions to medicines used in patients; and 9—Preparation of personalized sheets with the name and dosage of the medicine on the drug tray.

A five-step scale of risk assessment (from 1 to 5) was used to assess the above ergonomic factors: 1—minor risk, 2—little risk, 3—significant risk, 4—more significant risk, and 5—very significant risk.

The relationship between the levels of risk in the pharmacovigilance brochure and the likelihood of adverse health effects on the patient and the likelihood of a nurse/midwife being legally liable is shown in Table 1.

The NURIPH tool has been validated, and the Cronbach’s alpha coefficient for the NURIPH tool is 0.832. Based on the obtained value of the Cronbach’s alpha coefficient, it should be concluded that the tool is reliable. All items have positive discrimination power (Witczak et al., 2020) [15].

### 2.4. Statistical Analysis

The chi-squared test (with Yates’ correction for 2 × 2 tables) was used to compare qualitative variables among groups. In the case of low values in contingency tables, Fisher’s exact test was used instead. The Mann–Whitney U test was used to compare ordinal variables between two groups. The significance level for all statistical tests was set at 0.05. The analysis was performed in the R program, version 4.0.3 (R Core Team, Vienna, Austria, 2020) [16].

## 3. Results

### 3.1. The Characteristics of the Study Group 

The study involved 1774 nurses (1412 (79.6%) nurses from Poland and 362 (20.4%) nurses from Slovakia). Women prevailed in the study group—they accounted for 94.9% and 96.4% of the Polish and Slovak groups, respectively. Slovak nurses were younger—most of them, 38.4%, were in the age range of 40–49 years, and Polish nurses were predominantly (37.6%) in the age range of 50–59 years. A noticeably higher percentage of Polish nurses were graduates of master’s degree programs compared to Slovak nurses (54% of Polish and 38.7% of Slovak nurses). Regarding the professional experience of nurses in both countries, it has been found that the largest group of nurses had worked for more than 30 years (38.4% in Poland and 28.2% in Slovakia). Nurses from Poland most frequently carried out their work in large cities with populations of more than 500 thousand (30.3%), and nurses from Slovakia worked in cities with fewer than 50 thousand inhabitants (36.5%). More than 60% of Polish nurses and nearly 75% of Slovak nurses declared that they worked in no more than one place. The largest group of Polish nurses worked in medical treatment wards (27.8%), and most Slovak nurses worked in wards other than surgical and medical treatment wards (66.3%). The characteristics are presented in Table 2.

### 3.2. Results of the NURIPH Risk Matrix 

The results presented in Table 3 demonstrate that, in general, nurses in Poland assess the safety level of the pharmacotherapy process to be lower than nurses from Slovakia, i.e., they evaluate the existing risk factors as very significant. An analysis of individual elements of the risk matrix revealed that nurses from Slovakia most often, i.e., for six out of nine risk factors (items: one, five, six, seven, eight, and nine) assessed them as “significant risk (3)”, whereas Polish nurses most often, i.e., for as many as eight out of nine risk factors (items: one, two, three, four, five, six, seven, and nine) assessed risk factors as “very significant (5)”. Significant differences (*p* < 0.05) were demonstrated in eight (out of nine) risk factors. Only one risk factor was assessed very similarly by both groups, namely, the “limited availability of training on the effect of medicines, side effects and adverse reactions to medicines used in patients”—item 8, where *p* = 0.399. The mean score obtained in this case by Polish nurses was 3.62, and by Slovak nurses—3.54, which means that they assess this factor between “significant risk (3)” and “more significant risk (4)”. A certain similarity can be observed in the assessment of items two, three and four. Most Polish and Slovak nurses assessed the above-mentioned risks as “very significant risk (5)”, namely, inappropriate communication between doctors, nurses and midwifes regarding changes in drug orders, pressures of time during nurse/medical supervision, and inappropriate work organization.

Table 4 presents the results for questions from outside the NURIPH matrix. These questions asked about the safety culture in the organization where the nurses worked and the nurses’ opinions about the safety of the pharmacotherapy process and medication errors associated with it. Values for *p* below 0.05 indicated significant differences between the groups of nurses from Poland and Slovakia. Nurses from Poland assigned lower ratings to the overall level of pharmacotherapy safety at their work (*p* < 0.001). Moreover, they more frequently claim to be at the highest risk of committing errors (53%), and they less frequently claim that all persons involved are equally responsible for such errors, whereas Slovak nurses predominantly claim that all persons involved are equally responsible for such errors (82.3%) (*p* < 0.001). Another aspect in the questionnaire was the influence of electronic medical records on the elimination of risk factors in the pharmacotherapy process—nurses from Poland (42.5%) and Slovakia (48%) claimed that it will contribute to increasing the safety of pharmacotherapy, but only to a limited extent.

Both groups claimed that in the event of incorrect administration of a medicine, the patient affected or their family should be informed about such an occurrence (75.6% of Polish nurses and 69.6% of Slovak nurses). There was also a consistency in responses to the question as to who should inform the patients or their families about errors made in the pharmacotherapy process—Polish (62.7%) and Slovak (75%) nurses claimed that it is the attending physician or the doctor who issued the prescription who is responsible in this regard. Both in Poland and in Slovakia, the room where nurses prepare medications serves many purposes. Polish nurses, as a rule, inspect ward medicine stocks once or twice every six months (32.9%); Slovak nurses perform this task slightly more often, i.e., once or twice a month (28.2%). In both countries, regular training courses on the adverse effects of medications usually are provided in places other than the facilities in which nurses perform their work. They also have no possibility to have a consultation with a clinical pharmacologist. Regarding adverse events in the pharmacotherapy process related to the professional experience of nurses, an inverse correlation can be noted, i.e., nearly 70% Slovak nurses experienced them, and, on the other hand, 70% Polish nurses declared that they had not had such an experience (*p* < 0.001).

## 4. Discussion

Undoubtedly, in contemporary healthcare systems, there are a multitude of negative stressors that affect the safety of the pharmacotherapy process. They are related to such factors as nurse understaffing, population aging and the change in patient profile, patients with multiple morbidities, and state-of-the-art medical technologies, frequently with complex interfaces. These stressors place the frontline medical staff, such as nurses, in a situation in which they can fall short of their standards and be unable to provide the highest quality of care [17].

The authors’ own study has revealed that contemporary nurses are working under substantial time pressure (item three). This could result from the said shortages of personnel and excessive workloads. The consequences could affect the ability to ensure patient safety. Performing work under considerable pressure can be conducive to errors which might entail serious consequences in the pharmacotherapy process. Both Polish and Slovak nurses paid attention to this fact, and most of them, in both groups, considered working under time pressure as a “very significant risk (5)”.

In their work, El-Bannaat et al. emphasized that nurses should be supported in their provision of patient safety and also in their belief that errors in this process are predisposed by a great number of internal factors, such as shift pattern, and external factors, such as demographic or epidemiologic situations, and they should not, in the long run, entail the liability of nurses for incorrect pharmacotherapy [18].

A majority of both Polish and Slovak nurses assessed inappropriate work organization (item four) as a factor characterized by “very significant risk (5)”. In many medical facilities, the nurse who prepares medications for patients also performs other tasks, such as answering phone calls in the meantime, talking to another person who enters the room in which medications are prepared, etc. The pharmacotherapy process requires intense concentration, and the above-mentioned situations are conducive to medical errors. Moreover, it is rarely that separate rooms are assigned for medication preparation with access exclusively for the nurse appointed to that specific task. As demonstrated by research results, both in Poland and Slovakia, medicine preparation rooms serve many purposes. The preparation of personalized sheets with the name and dosage of the medicine on the drug tray poses an additional problem which has been assessed by Polish nurses as a “very significant risk (5)”, and by Slovak nurses as a “significant risk (3)”.

Studies conducted in Iran on errors in the pharmacotherapy process in teaching hospitals have revealed that the majority of factors are related to the work of nurses, for example, insufficient care of patient medical records, dissatisfaction with work, and errors in the calculation of medication doses [19]. These studies are noteworthy, because in 2007, the frequency of errors in pharmacotherapy in Iran was around 2.4–5.6 times higher than in the United States [20].

Polish nurses also paid attention to the legibility of medical documentation as far as medical orders are concerned (item one). They assessed this factor as a “very significant risk (5)”. In Slovakia, in turn, it was assessed as a “significant risk (3)”. This might result, among other reasons, from the fact that medical documents in Slovakia are already in electronic formats, whereas in Poland, not all medical institutions had implemented this system at the time of conducting this study. Legibility of documentation (especially of medical orders) and medical order deficiencies are other factors that play a significant role in ensuring patient safety. Not infrequently, medical orders fail to provide information on the specific solvent for a particular medicine (item six). Polish nurses consider it as a “very significant risk (5)”, and Slovak nurses as a “significant risk (3)”.

Bryant R. et al. emphasize that the education of nurses by pharmacists/clinical pharmacologists on the use of some groups of medications, in particular of those from the high-risk group, i.e., narcotics, medications used in cancer treatment, medications for heart diseases, etc., could contribute to reducing errors in the pharmacotherapy process [21].

Unfortunately, the authors’ own research has demonstrated that neither in Poland nor in Slovakia is there any possibility for nurses to consult a hospital pharmacist/clinical pharmacologist. Moreover, nurses from Poland drew attention to the fact that they are not familiar with the list of generic medications, and assesses this factor (item five) as “very significant risk (5)”; Slovak nurses assessed it as “significant risk (3)”. Knowledge deficiencies in safe pharmacotherapy could be eliminated by staff training courses. At the same time, nurses from Poland and Slovakia stated that they are not trained on a regular basis, and most of them assessed the factor of the availability of training (item eight) as “significant risk (4)”.

The absence of consultation possibilities is a symptom of lacking communication. Communication in a therapeutic team, as emphasized by Soodabeh et al., is of crucial importance [22]. Both Polish and Slovak nurses stated that inappropriate communication in the therapeutic team is a significant risk factor (item two).

Keers R.N. et al., in their systematic review of literature on medication administration errors (MAEs) in hospital, noted that many system-related factors contribute to errors in the pharmacotherapy process. It is important not only to differentiate between the types of these factors, but also to analyze how they are generated and how they combine to ultimately result in harm to patients. It is therefore of special importance that research on the safety of pharmacotherapy is conducted at both national and international scales with regard to particular healthcare systems [23].

Ensuring patient safety is an immensely difficult task, as demonstrated both by the authors’ own study and studies conducted by other researchers. Nevertheless, it is an important task not only for medical staff but also for healthcare managers.

### Limitations of the Study

The study group was feminized, with nearly 95% consisting of women, which is because, for years, nursing staff have primarily comprised women in Poland and Slovakia. This gender distribution in the study group makes it impossible to compare the results in both genders: women versus men. It can be assumed that there are differences in risk estimation in the pharmacotherapy process between female nursing staff and male nursing staff. The study indicates that women differ from men in their perception of different elements of the environment, which could be reflected in the results of the study with an equal distribution in terms of gender. However, this is significant due to the fact that there are men among the nursing staff.

## 5. Conclusions

The literature review and the results of the authors’ own study demonstrate that the pharmacotherapy process implemented by nurses, regardless of the country, is subject to numerous risk factors and can adversely affect patient safety. Staff deficiencies, working under time pressure, excessive responsibilities, inadequate work organization, or disturbed interpersonal communication in a medical team can lead to medication errors, which can ultimately result in patient death. It has been found that the safety level of pharmacotherapy is assessed by Polish nurses as much lower than by Slovak nurses. The needs for work reorganization continue to be ignored, and they include, at least, assigning a room to be used exclusively to carry out medical orders, and accessible only to those involved in the pharmacotherapy process. In summary, educational activities dedicated to the entire medical staff involved in the pharmacotherapy process should be implemented in the field of adverse events, adverse effects of medications, and appropriate and effective communication. Managers, in turn, should be made aware that changing work organization, improving work conditions, and avoiding accusations and searching for persons to blame in a situation of an adverse event can considerably improve patient safety in their organizations.

## Figures and Tables

**Table 1:** Linking risk levels in the pharmacotherapy process to negative health consequences for the patient and the legal responsibility of nurses and midwives.

Level of Risk	Probability of Adverse Health Effects for the Patient Resulting from Medication Errors	Probability of Legal Liability for the Nurse/Midwife Resulting from Medication Errors
Minor risk (1)	Low probability	Low probability
Little risk (2)	Unlikely	Unlikely
Significant risk (3)	Probable	Probable
More significant risk (4)	Likely	Likely
Very significant risk (5)	Very likely	Very likely

**Note:** Based on Witczak at al., 2020 [15].

**Table 2:** Characteristics of the studied group of nurses.

Parameter		Country		*p*
		Poland (*n* = 1412)	Slovakia (*n* = 362)	
Profession	Nurse	1412 (100%)	362 (100%)	*p* = 0.18
Gender	Female	1340 (94.90%)	349 (96.41%)	*p* = 0.289
	Male	72 (5.10%)	13 (3.59%)	
Age	20–29 years	190 (13.46%)	58 (16.02%)	*p* < 0.001 *
	30–39 years	175 (12.39%)	63 (17.40%)	
	40–49 years	428 (30.31%)	139 (38.40%)	
	50–59 years	531 (37.61%)	83 (22.93%)	
	60 years or more	88 (6.23%)	19 (5.25%)	
Education	Secondary	223 (15.79%)	75 (20.72%)	*p* < 0.001 *
	Bachelor’s degree	367 (25.99%)	108 (29.83%)	
	Master’s degree	772 (54.67%)	140 (38.67%)	
	Doctorate	26 (1.84%)	8 (2.21%)	
	Other	24 (1.70%)	31 (8.56%)	
Work experience	Up to 5 years	187 (13.24%)	71 (19.61%)	*p* < 0.001 *
	6–10 years	116 (8.22%)	32 (8.84%)	
	11–19 years	136 (9.63%)	56 (15.47%)	
	20–29 years	431 (30.52%)	101 (27.90%)	
	30 years or more	542 (38.39%)	102 (28.18%)	
Size of the town in which work is performed	Rural area	53 (3.75%)	21 (5.80%)	*p* < 0.001 *
	City of up to 50 thousand inhabitants	344 (24.36%)	132 (36.46%)	
	City of 50–100 thousand inhabitants	193 (13.67%)	116 (32.04%)	
	City of 100–500 thousand inhabitants	394 (27.90%)	64 (17.68%)	
	City of over 500 thousand inhabitants	428 (30.31%)	29 (8.01%)	
Work in more than one place	Yes	548 (38.81%)	93 (25.69%)	*p* < 0.001 *
	No	864 (61.19%)	269 (74.31%)	
Ward	Surgical ward	347 (24.58%)	63 (17.40%)	*p* < 0.001 *
	Medical treatment ward	393 (27.83%)	59 (16.30%)	
	Manager positions	383 (27.12%)	0 (0.00%)	
	Other	289 (20.47%)	240 (66.30%)	

* Statistically significant (*p* < 0.05).

**Table 3:** Results for individual risk factors in pharmacotherapy in Polish and Slovak nurses.

Item	Country	Minor Risk (1)	Little Risk (2)	Significant Risk (3)	More Significant Risk (4)	Very Significant Risk (5)	Mean	SD	*p*
Item 1: Poorly legible or illegible medical orders	Poland	42 (2.97%)	19 (1.35%)	174 (12.32%)	138 (9.77%)	1039 (73.58%)	4.50	0.97	*p* < 0.001 *
	Slovakia	30 (8.29%)	29 (8.01%)	137 (37.85%)	51 (14.09%)	115 (31.77%)	3.53	1.24	
Item 2: Inappropriate communication between physician, nurse, and midwife regarding changes in drug orders	Poland	40 (2.83%)	31 (2.20%)	229 (16.22%)	295 (20.89%)	817 (57.86%)	4.29	1.00	*p* < 0.001 *
	Slovakia	37 (10.22%)	24 (6.63%)	111 (30.66%)	71 (19.61%)	119 (32.87%)	3.58	1.28	
Item 3: Time pressure during nurse/medical supervision	Poland	19 (1.35%)	40 (2.83%)	273 (19.33%)	286 (20.25%)	794 (56.23%)	4.27	0.96	*p* = 0.008 *
	Slovakia	5 (1.38%)	16 (4.42%)	84 (23.20%)	80 (22.10%)	177 (48.90%)	4.13	1.00	
Item 4: Inappropriate work organization: preparation of medicines for patients combined with the simultaneous performance of other activities by a nurse/midwife (for example, answering calls, execution of current diagnostic orders, etc.)	Poland	26 (1.84%)	36 (2.55%)	174 (12.32%)	232 (16.43%)	944 (66.86%)	4.44	0.93	*p* < 0.001 *
	Slovakia	7 (1.93%)	20 (5.52%)	95 (26.24%)	87 (24.03%)	153 (42.27%)	3.99	1.04	
Item 5: Lack of clarity or illegibility of medical orders for nurses and midwives	Poland	91 (6.44%)	112 (7.93%)	384 (27.20%)	314 (22.24%)	511 (36.19%)	3.74	1.21	*p* < 0.001 *
	Slovakia	38 (10.50%)	44 (12.15%)	122 (33.70%)	63 (17.40%)	95 (26.24%)	3.37	1.28	
Item 6: No physician’s prescription of a specific solvent for a particular medicine	Poland	128 (9.07%)	229 (16.22%)	381 (26.98%)	290 (20.54%)	384 (27.20%)	3.41	1.29	*p* = 0.004 *
	Slovakia	42 (11.60%)	50 (13.81%)	137 (37.85%)	62 (17.13%)	71 (19.61%)	3.19	1.23	
Item 7: Shift work causing psycho-physiological fatigue;	Poland	63 (4.46%)	127 (8.99%)	422 (29.89%)	297 (21.03%)	503 (35.62%)	3.74	1.16	*p* = 0.017 *
	Slovakia	15 (4.14%)	44 (12.15%)	118 (32.60%)	81 (22.38%)	104 (28.73%)	3.59	1.15	
Item 8: Limited availability of training on the effects of medicines, side effects and adverse reactions to medicines used in patients	Poland	62 (4.39%)	153 (10.84%)	457 (32.37%)	330 (23.37%)	410 (29.04%)	3.62	1.14	*p* = 0.399
	Slovakia	27 (7.46%)	30 (8.29%)	123 (33.98%)	83 (22.93%)	99 (27.35%)	3.54	1.19	
Item 9: Preparation of personalized sheets with the name and dosage of the medicine on the drug tray	Poland	151 (10.69%)	191 (13.53%)	366 (25.92%)	241 (17.07%)	463 (32.79%)	3.48	1.35	*p* < 0.001 *
	Slovakia	49 (13.54%)	59 (16.30%)	137 (37.85%)	48 (13.26%)	69 (19.06%)	3.08	1.26	

*P*—Mann–Whitney test; * statistically significant (*p* < 0.05).

**Table 4:** Comparison of the results from outside the risk matrix for the group of nurses from Poland and Slovakia.

Parameter		Country		*p*
		Poland (*n* = 1412)	Slovakia (*n* = 362)	
In your opinion, what is the overall safety level of the pharmacotherapy process in your employing organization?	Low	172 (12.18%)	24 (6.63%)	*p* < 0.001 *
	Average	783 (55.45%)	178 (49.17%)	
	High	420 (29.75%)	146 (40.33%)	
	I do not know	37 (2.62%)	14 (3.87%)	
In your opinion, who is at the highest risk of committing errors in the implementation of the pharmacotherapy process?	Physician	49 (3.47%)	29 (8.01%)	*p* < 0.001 *
	Nurse, midwife	750 (53.12%)	33 (9.12%)	
	Hospital pharmacy employee	4 (0.28%)	2 (0.55%)	
	They are all equally responsible	609 (43.13%)	298 (82.32%)	
In your opinion, will electronic medical records eliminate risk factors in the pharmacotherapy process?	Yes	410 (29.04%)	35 (9.67%)	*p* < 0.001 *
	No	283 (20.04%)	95 (26.24%)	
	To a limited extent	600 (42.49%)	174 (48.07%)	
	I don’t know	119 (8.43%)	58 (16.02%)	
Do you think that in the event of incorrect administration of a medicine, the patient affected or their family should be informed about such an occurrence?	Yes	1071 (75.85%)	252 (69.61%)	*p* = 0.004 *
	No	131 (9.28%)	55 (15.19%)	
	It doesn’t really matter	210 (14.87%)	55 (15.19%)	
In your opinion, who should inform the patients or their families about errors committed in the pharmacotherapy process?	Head/manager of the ward	288 (20.40%)	66 (18.23%)	*p* < 0.001 *
	Attending physician/doctor who issued the prescription	885 (62.68%)	272 (75.14%)	
	Nurse/midwife carrying out the order	108 (7.65%)	3 (0.83%)	
	Patient ombudsman	60 (4.25%)	15 (4.14%)	
	Another person	71 (5.03%)	6 (1.66%)	
The room in which medications are prepared for patients is:	A room serving only this particular purpose	269 (19.05%)	69 (19.06%)	*p* = 0.001 *
	A room serving many purposes	569 (40.30%)	181 (50.00%)	
	A room which is also used to perform medical procedures	574 (40.65%)	112 (30.94%)	
At your workplace, supervision of medicines is carried out:	During day/morning shifts	398 (28.19%)	62 (17.13%)	*p* < 0.001 *
	During night shifts	374 (26.49%)	122 (33.70%)	
	During all shifts	640 (45.33%)	178 (49.17%)	
How often do pharmacy employees carry out inspections of ward medicine stocks?	Daily	6 (0.42%)	3 (0.83%)	*p* < 0.001 *
	Once or twice per week	23 (1.63%)	13 (3.59%)	
	Once or twice per month	385 (27.27%)	102 (28.18%)	
	Once or twice per quarter	323 (22.88%)	36 (9.94%)	
	Once or twice every six months	465 (32.93%)	95 (26.24%)	
	Once or twice per year	70 (4.96%)	9 (2.49%)	
	Never	122 (8.64%)	87 (24.03%)	
	Unknown	18 (1.27%)	17 (4.70%)	
Are there internal training courses on adverse effects of medications held at your workplace on a regular basis?	Yes	224 (15.86%)	24 (6.63%)	*p* < 0.001 *
	No	716 (50.71%)	252 (69.61%)	
	Occasionally	356 (25.21%)	72 (19.89%)	
	Only after adverse drug reaction occurs	116 (8.22%)	14 (3.87%)	
At your workplace, is there any possibility to consult a clinical pharmacologist at the time of preparing drug orders and administering medicines to patients?	Yes	158 (11.19%)	106 (29.28%)	*p* < 0.001 *
	No	1020 (72.24%)	176 (48.62%)	
	To a limited extent	234 (16.57%)	80 (22.10%)	
In your professional practice, have you experienced any adverse events in the pharmacotherapy process?	Yes	437 (30.95%)	250 (69.06%)	*p* < 0.001 *
	No	975 (69.05%)	112 (30.94%)	

*P*—Mann–Whitney test for quantitative variables, chi-squared or Fisher’s exact test for qualitative variables; * Statistically significant (*p* < 0.05).

## Data Availability

Data sharing not applicable.
